# Central composite design for the development of carvedilol-loaded transdermal ethosomal hydrogel for extended and enhanced anti-hypertensive effect

**DOI:** 10.1186/s12951-021-00833-4

**Published:** 2021-04-09

**Authors:** Padmanabha Rao Amarachinta, Garima Sharma, Noufel Samed, Ananda Kumar Chettupalli, Madhusudhan Alle, Jin-Chul Kim

**Affiliations:** 1 School of Pharmacy, Anurag University, Telangana, 500088 India; 2 Department of Biomedical Science & Institute of Bioscience and Biotechnology, Kangwon National University, Chuncheon, 24341 Republic of Korea

**Keywords:** Ethosomes, Carvedilol, Central composite design (CCD), Ethosomal gel, Anti-hypertension

## Abstract

**Background:**

Carvedilol, the anti-hypertensive drug, has poor bioavailability when administered orally. Ethosomes-mediated transdermal delivery is considered a potential route of administration to increase the bioavailability of carvedilol. The central composite design could be used as a tool to optimize ethosomal formulation. Thus, this study aims to optimize carvedilol-loaded ethosomes using central composite design, followed by incorporation of synthesized ethosomes into hydrogels for transdermal delivery of carvedilol.

**Results:**

The optimized carvedilol-loaded ethosomes were spherical in shape. The optimized ethosomes had mean particle size of 130 ± 1.72 nm, entrapment efficiency of 99.12 ± 2.96%, cumulative drug release of 97.89 ± 3.7%, zeta potential of − 31 ± 1.8 mV, and polydispersity index of 0.230 ± 0.03. The *in-vitro* drug release showed sustained release of carvedilol from ethosomes and ethosomal hydrogel. Compared to free carvedilol-loaded hydrogel, the ethosomal gel showed increased penetration of carvedilol through the skin. Moreover, ethosomal hydrogels showed a gradual reduction in blood pressure for 24 h in rats.

**Conclusions:**

Taken together, central composite design can be used for successful optimization of carvedilol-loaded ethosomes formulation, which can serve as the promising transdermal delivery system for carvedilol. Moreover the carvedilol-loaded ethosomal gel can extend the anti-hypertensive effect of carvedilol for a longer time, as compared to free carvedilol, suggesting its therapeutic potential in future clinics.
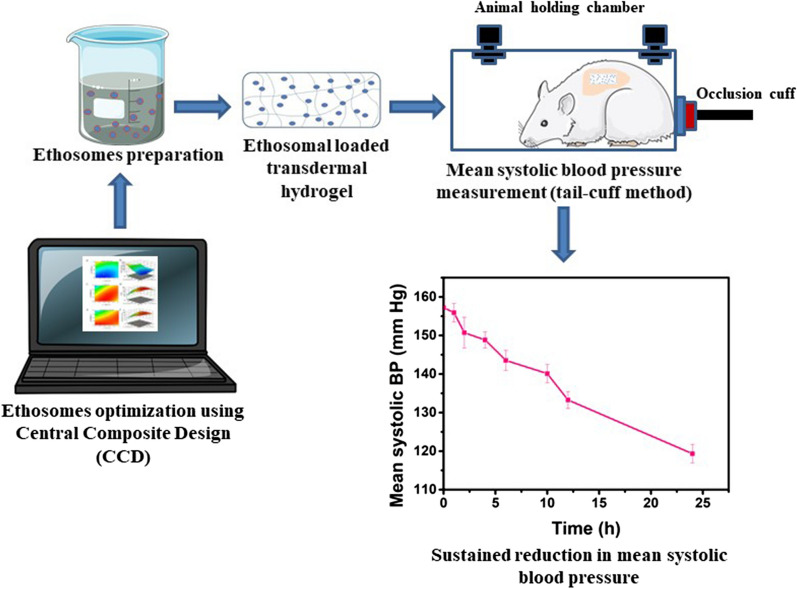

**Supplementary Information:**

The online version contains supplementary material available at 10.1186/s12951-021-00833-4.

## Background

Transdermal drug delivery is advantageous over other established routes of delivery, because unlike oral route, the transdermal delivery prevents the drug from pre-systemic metabolism which results in better bioavailability. Moreover, the transdermally-administered drugs can evade enzymatic degradation and offers a non-invasive route for drug administration, thus providing better patient compliance [1]. However, the primary challenge for the transdermal delivery of drugs is crossing tightly connected stratum corneum (SC) barrier, which limits drug permeation [2]. To improve transdermal drug penetration, various physical and chemical methods have been reported, including iontophoresis, microneedles and nanocarriers, such as liposomes and nanolipid particles [3].

Carvedilol is a popular drug used in the treatment of hypertension and cardiovascular disorders [4]. Although carvedilol is well accepted in clinics, it undergoes first-pass metabolism and has low oral bioavailability (about 25% to 35%) [5]. The low oral bioavailability of carvedilol is possibly because of its low dissolution capability (ranked in the class II category of biopharmaceutical classification system), and its profound pre-systemic metabolism [6, 7]. Therefore, there is a need to develop an alternative method for carvedilol delivery, such as transdermal delivery. Carvedilol is ideal for transdermal delivery because of its high lipophilicity and low molecular weight (406.5 g/ml) [8].

In the last few years, several transdermal patches have been developed for the delivery of carvedilol through the skin. Carvedilol has been delivered through various transdermal delivery systems, such as nanoemulsions [9], lipid based nanoparticles [10], and matrix based systems [11]. A matrix based transdermal patch showed enhanced bioavailability of carvedilol by 72%, as compared to the oral route [11]. However, these drug delivery systems might have certain disadvantages, such as complex formulation and high cost.

Ethosomes are lipid based vesicular drug carriers which consists of high ethanol concentration. The high concentration of ethanol in ethosomes imparts them the ability to modify the highly dense alignment of the lipid bilayers in the SC, thereby ensuring deeper drug penetration [12]. The presence of ethanol also impart a net negative on the surface of ethosomes that provide better stability due to electrostatic repulsion [13]. The high concentration of ethanol also ensures high solubility of lipophilic drugs thereby increasing the entrapment efficiency (EE) [14]. Moreover, the ethosomes are less toxic and cause less skin irritation hence making ethosomes suitable for transdermal delivery [15].

Studies have shown that the incorporation of vesicular carriers into hydrogel can improve their stability and skin penetration ability [16]. The hydrogels are known to be highly compatible with ethosomal formulations and provides better bio-adhesive properties rendering favourable conditions for transdermal drug delivery [17]. Ibrahim et al. (2018) had developed an ethosomal hydrogel drug delivery system for carvedilol using hydroxypropyl methylcellulose (HPMC) as a thickening agent [18]. They showed that the incorporation of carvedilol-loaded ethosomes (CLE) into a hydrogel helped in achieving a sustained release of carvedilol hence providing a longer anti-hypertensive effect, as compared to its free form [18].

It was observed that when the concentration of ethanol exceeds beyond a certain limit during the synthesis of ethosomes, it makes the vesicular membrane leaky which can result in low EE, and reduced stability for ethosomes [19, 20]. Hence, optimal formulation of ethosomes is suggested for the synthesis of ethosomes with better physicochemical characteristics. For proper optimization of ethosomal formulations statistical design studies have to be conducted under a given set of conditions. Based on the currently available experimental design methods, the central composite design (CCD), is very much preferred [21]. The CCS is a robust form of surface response methodology (SRM) [22], which evaluates the extent of influence of many individual variables involved in an experiment [23, 24]. The optimization procedure is done using the Design of experiments (DoE) software which consists of choosing the critical variables, formulating mathematical equations, and running a set number of trials by giving limits to the chosen variables [24, 25]. The contour response graphs are then plotted to analyse the correlation among the selected variables and thereby determine the appropriate experimental conditions for the best possible formulation [24, 25]. Hence, CCD can be used to optimize the preparation of ethosomes with minimum experimental trials.

This study aimed to optimize the synthesis of CLE suspension using CCD model. Moreover, this study also aimed to enhance the drug permeation and pharmacological effect of carvedilol by incorporating the optimized ethosomal suspension into hydrogel for transdermal delivery. Further, the purpose of this study is to control the rate of carvedilol delivery across the skin to extend the anti-hypertensive effect of the drug for a longer period of time.

## Results

### Solubility profile of carvedilol

The permeation and efficacy of carvedilol delivered through the transdermal route are highly dependent on the extent of its solubility in ethosomal suspension. The solubility of carvedilol might also influence, ethosomal vesicle size, drug EE, and cumulative drug release (CDR) from the ethosomes. Here, we attempted to find a suitable dispersion medium for carvedilol for the synthesis of ethosomal suspension. It was observed that the solubility of carvedilol increases with an increase in the concentration of ethanol, as seen in Table 1.

**Table 1:** Solubility studies of carvedilol in different media (Mean ± S.D, n = 3)

S. no	Medium composition (Ethanol: D/W) % (v/v)	Solubility (mg/ml)
1	20:80	0.22 ± 0.01
2	30:70	0.34 ± 0.08
3	40:60	0.57 ± 0.06
4	50:50	0.76 ± 0.11

### Optimization of carvedilol-loaded ethosome (CLE) formulation using CCD

A rotatable CCD was used around a fixed point for preparing CLE suspensions [26]. In this study, the independent variables (X_i_) taken into consideration were phospholipid % (w/v) (X_1_), ethanol % (v/v) (X_2_), and propylene glycol (PG) % (v/v) (X_3_). The range decided for each independent variables are shown in Table 2. The dependant variables (Y_i_) considered were vesicle size (Y_1_), % EE (Y_2_), and % CDR (Y_3_). The desired response for each dependent variable can be seen in Table 3.

**Table 2:** The upper and lower limits of the independent variables for CCD

Independent variables	Levels		
	Low	Medium	High
X_1_ (Lipid) % (w/v)	2	3.5	5
X_2_ (Ethanol) % (v/v)	20	30	40
X_3_ (PG) % (v/v)	5	7.5	10

**Table 3:** The desired response of the dependant variables for CCD

Dependent variable (response)	Desirability constraints
Y_1_ (Vesicle size (nm))	Minimize
Y_2_ (% EE)	Maximize
Y_3_ (% CDR)	Maximize

The response surface methodology (RSM) contour surface plots show the correlation between the dependent and independent variables, which were constructed by keeping one of the factors at a constant level. In this study, the variations in the vesicle size (Y_1_), % EE (Y_2_), and % CDR (Y_3_) were observed by varying the concentrations of phospholipid (X_1_) and ethanol (X_2_), while keeping the concentration of PG (X_3_) constant at 7.5% (v/v).

**Fig. 1:**
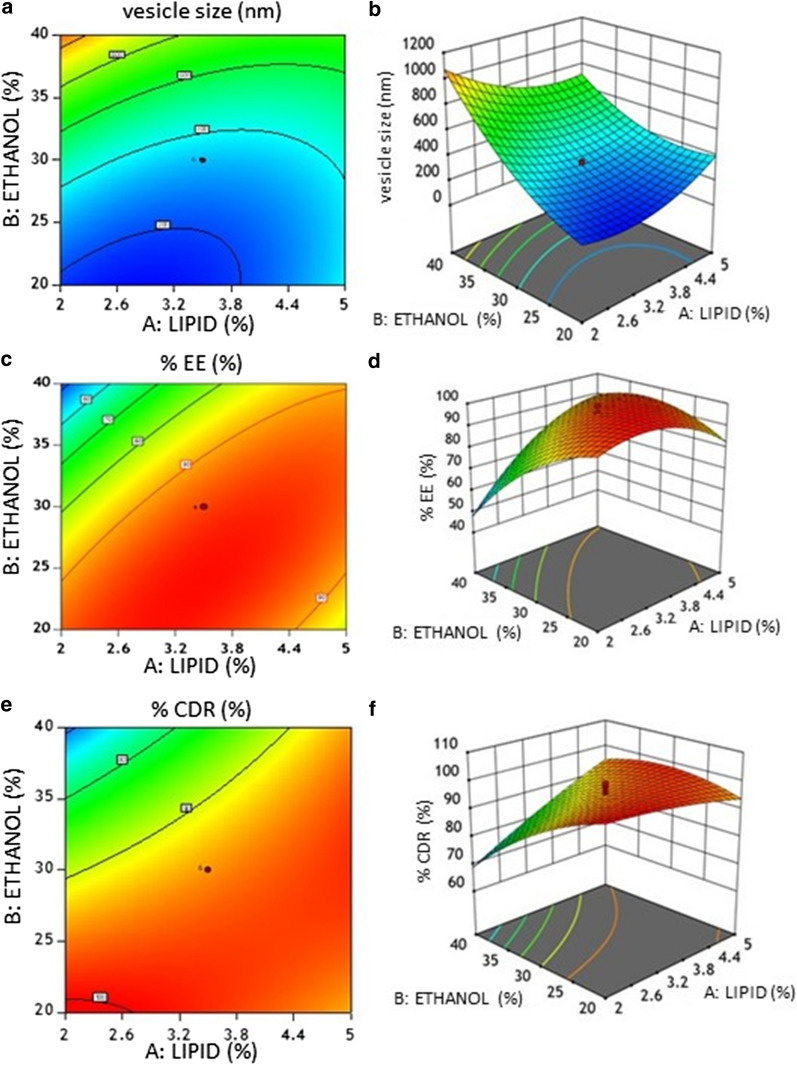
Contour plots of responses showing the interactive effects of amount of lipid and amount of ethanol on vesicle size (Y_1_) (**a**, **b**), % EE (Y_2_) (**c, d**), and % CDR (Y_3_) (**e**, **f**)

According to Table 4, EF1–EF20 formulations showed wide variations in vesicle size, ranging from around 130 nm to 1200 nm (Table 4). It can be seen that the vesicle size strongly depends on the selected variables. From RSM data shown in Fig. 1a, b, it can be inferred that the vesicle size decreases when the concentration of phospholipid is increased for all the levels of ethanol concentrations.

**Table 4:** Composition and Characterization of carvedilol ethosomal formulations

Independent variables				Dependent variables				
Formulation Code (EF)	A: Lipid % (w/v) (X_1_)	B: Ethanol % (v/v) (X_2_)	C: Propylene glycol % (v/v) (X_3_)	Vesicle Size (nm) (Y_1_)	% EE (Y_2_)	% CDR (Y_3_)	PDI	Zeta Potential (mV)
1	2	20	5	130 ± 1.72	99.12 ± 2.96	97.89 ± 3.7	0.230 ± 0.03	− 31 ± 1.8
2	3.5	13.9	7.5	280 ± 1.89	89.56 ± 2.35	95.87 ± 3.5	0.272 ± 0.05	− 29 ± 1.56
3	5	40	10	600 ± 1.96	94.09 ± 2.89	98.74 ± 3.6	0.264 ± 0.09	− 32 ± 1.46
4	5	20	10	200 ± 1.51	95.82 ± 2.91	98.82 ± 3.6	0.281 ± 0.31	− 34 ± 0.45
5	3.5	30	3.3	550 ± 2.82	94.41 ± 2.87	87.5 ± 2.8	0.254 ± 0.46	− 41 ± 0.12
6	3.5	30	7.5	321 ± 1.94	95.24 ± 2.90	93.35 ± 3.1	0.157 ± 0.43	− 35 ± 0.46
7	3.5	46.8	7.5	1200 ± 2.95	52.67 ± 1.58	71.08 ± 1.7	0.238 ± 0.91	− 29 ± 0.59
8	5	20	5	550 ± 2.82	71.82 ± 1.98	88.23 ± 2.7	0.268 ± 0.06	− 28 ± 0.99
9	3.5	30	7.5	345 ± 1.51	98.87 ± 2.89	92.52 ± 3.0	0.312 ± 0.01	− 29 ± 1.17
10	3.5	30	7.5	315 ± 1.42	94.25 ± 2.87	90.58 ± 2.8	0.170 ± 0.03	− 31 ± 1.15
11	3.5	30	7.5	318 ± 1.42	95.46 ± 2.88	98 ± 3.6	0.135 ± 0.04	− 34 ± 1.25
12	2	40	5	1050 ± 2.01	56.74 ± 1.65	69.29 ± 1.2	0.235 ± 0.09	− 39 ± 0.48
13	0.97	30	7.5	800 ± 1.91	55.45 ± 1.62	85.26 ± 2.4	0.240 ± 0.01	− 41 ± 1.23
14	3.5	30	7.5	311 ± 1.95	95.78 ± 2.89	99.24 ± 3.8	0.235 ± 0.05	− 44 ± 0.85
15	6	30	7.5	632 ± 1.92	81.79 ± 1.98	98.51 ± 3.4	0.421 ± 0.06	− 40 ± 1.23
16	2	20	10	130 ± 1.72	99.08 ± 2.96	99.89 ± 3.9	0.123 ± 0.01	− 37 ± 0.90
17	3.5	30	7.5	350 ± 1.54	93.56 ± 2.77	96.07 ± 3.5	0.284 ± 0.05	− 36 ± 1.45
18	5	40	5	868 ± 1.98	90.53 ± 2.21	89.28 ± 3.1	0.184 ± 0.46	− 28 ± 0.85
19	2	40	10	1086 ± 2.01	44.82 ± 1.38	64.89 ± 0.9	0.294 ± 0.09	− 29 ± 0.34
20	3.5	30	11.7	190 ± 1.86	98.01 ± 2.85	99 ± 3.9	0.431 ± 0.13	− 27 ± 1.22

The % EE of a drug is an essential parameter to estimate the amount of the drug-loaded in any drug delivery system. It helps in assessing the suitability of a drug delivery system to encapsulate the concerned drug. Similar to vesicle size, the % EE results also showed a wide range, from around 44% to 99% (Table 4). It was observed that at different levels of ethanol concentrations, the increase in the concentrations of phospholipid resulted in the increase of % EE. Increasing the concentration of ethanol from 15 to 45% (v/v) increases the % EE. However, a further increase in the ethanol concentration (> 45% (v/v)) decreases % EE, possibly due to solubilization of the phospholipids.

The determination of % CDR is important to evaluate the release of drug from optimized ethosomal formulations prior to pharmacological testing [27]. Here, the % CDR ranged from around 65% to 99% in all the formulations (Table 4). From Fig. 1e, f, it can be seen that % CDR increased with increase an in phospholipids concentrations for different levels of ethanol concentrations. This variation of % CDR showed a trend similar to what was observed for % EE. Therefore, it can be suggested that the vesicle size, % EE, and % CDR of ethosomes are highly dependent on the concentrations of phospholipid, ethanol, and PG.

The polydispersity index (PDI) values of less than 0.5 is an indicator of the homogenous size distribution of vesicles [28]. In this study, the PDI values for all the ethosomal formulations (i.e. EF1 – EF20) were less than 0.45 (Table 4), suggesting the possibility of uniform size distribution in all the simulated ethosomal formulations. Further, it was also observed that all the formulations had a negative zeta potential value within the range of − 27.81 to − 44.51 mV. Zeta potential is a critical factor that affects the stability and skin permeation ability of ethosomes [29]. The high positive or negative zeta potential values (more than ± 30 mV) are expected to deliver a strong electrostatic repulsion and prevent the aggregation of similarly charged nanoparticles in the dispersion [30].

The regression equations obtained for Y_1_, Y_2_, and Y_3_ are as follows:

1 $${\mathrm{Y}}_{1}= 328.21-34.45{\mathrm{X}}_{1}+302.50{\mathrm{X}}_{2}-87.68{\mathrm{X}}_{3}-143.50{\mathrm{X}}_{1}{\mathrm{X}}_{2}- 80.50 {\mathrm{X}}_{1}{\mathrm{X}}_{3}+16.00 {\mathrm{X}}_{2}{\mathrm{X}}_{3}+127.57 {\mathrm{X}}_{1}^{2}+136.05 {\mathrm{X}}_{2}^{2}+5.24 {\mathrm{X}}_{3}^{2}$$

2 $${\mathrm{Y}}_{2}= 95.43+7.59{\mathrm{X}}_{1}-9.87{\mathrm{X}}_{2}+2.15{\mathrm{X}}_{3}+13.21{\mathrm{X}}_{1}{\mathrm{X}}_{2}+4.10 {\mathrm{X}}_{1}{\mathrm{X}}_{3}-4.88 {\mathrm{X}}_{2}{\mathrm{X}}_{3}- 8.90{\mathrm{X}}_{1}^{2}-8.90 {\mathrm{X}}_{2}^{2}+0.8494 {\mathrm{X}}_{3}^{2}$$

3 $${\mathrm{Y}}_{3}= 94.98+4.71{\mathrm{X}}_{1}-7.72{\mathrm{X}}_{2}+2.63{\mathrm{X}}_{3}+8.21{\mathrm{X}}_{1}{\mathrm{X}}_{2}+2.95 {\mathrm{X}}_{1}{\mathrm{X}}_{3}-0.8025 {\mathrm{X}}_{2}{\mathrm{X}}_{3}- 1.23{\mathrm{X}}_{1}^{2}-4.21{\mathrm{X}}_{2}^{2}-0.7502$$

where, X_1_, X_2_ and X_3_ are the independent variables % (w/v) Phospholipid, % (v/v) Ethanol, and % (v/v) PG respectively. Y_1_, Y_2,_ and Y_3_ are the dependent variables vesicle size (nm), % EE, and % CDR, respectively.

In the regression equations, the sign and the value associated with each variable represent the tendency and magnitude of the terms influencing the responses. From the regression equations, it was observed that a quadratic relation between the dependent and independent variables is more suitable with a better correlation among the variables, as shown in Table 5. To imply a good and effective correlation, the R^2^ value should be at least 0.80. The observed R^2^ $({\mathrm{R}}_{\mathrm{O}}^{2})$ values of Y_1_, Y_2_ and Y_3_ are 0.9890, 0.9887, and 0.9662, respectively. The adjusted R^2^ $({\mathrm{R}}_{\mathrm{A}}^{2})$ values of Y_1_, Y_2_ and Y_3_ are 0.9790, 0.9785, and 0.9358, respectively, which were high enough to indicate the significance of the model (Table 5). The predicted R^2^ $({\mathrm{R}}_{\mathrm{P}}^{2})$ values of Y_1_, Y_2_ and Y_3_ are 0.9203, 0.9318, and 0.9025, respectively, which indicated a good correlation between the predicted and observed values.

**Table 5:** Regression values of the selected responses during optimization

Model	Y_1_			Y_2_			Y_3_		
	${\mathrm{R}}_{\mathrm{O}}^{2}$	${\mathrm{R}}_{\mathrm{A}}^{2}$	${\mathrm{R}}_{\mathrm{P}}^{2}$	${\mathrm{R}}_{\mathrm{O}}^{2}$	${\mathrm{R}}_{\mathrm{A}}^{2}$	${\mathrm{R}}_{\mathrm{P}}^{2}$	${\mathrm{R}}_{\mathrm{O}}^{2}$	${\mathrm{R}}_{\mathrm{A}}^{2}$	${\mathrm{R}}_{\mathrm{P}}^{2}$
Linear	0.6611	0.5976	0.4484	0.3675	0.2489	0.0766	0.5597	0.4771	0.2359
2FI	0.7666	0.6588	0.5264	0.6574	0.4993	0.3302	0.8434	0.7711	0.6347
Quadratic	0.9890	0.9790	0.9203	0.9887	0.9785	0.9318	0.9662	0.9358	0.9025
*p* value	0.0001			0.0001			0.0001		

To further substantiate the results, Analysis of variance (ANOVA) analysis was performed and higher F- values obtained for each variable shows that the models were well-suited for optimizing the experimental conditions. ANOVA analysis also indicates that the quadratic regression model was significant and valid for each of the responses Y_1_ (p < 0.0001), Y_2_ (p < 0.0001) and Y_3_ (p < 0.0001) (Table 6).

**Table 6:** ANOVA of optimized quadratic model of the novel ethosomal formulation

Parameter	Source	DF	Sum of squares	Mean of squares	F Value	*p* Value
Y_1_ (Vesicle size)	Model	9	2.051	2.279	99.65	< 0.0001
	Residual	10	22,866.43	2286.64		
	Lack of fit	5	21,497.1	4299.42	15.69	0.0045
	Pure error	5	1369.33	273.87		
Y_2_ (% EE)	Model	9	5869.50	652.17	97.24	< 0.0001
	Residual	10	67.07	6.71		
	Lack of fit	5	50.24	10.05	2.98	0.0029
	Pure error	5	16.83	3.37		
Y_3_ (% CDR)	Model	9	2090.19	232.24	31.76	< 0.0001
	Residual	10	73.13	7.31		
	Lack of fit	5	16.61	3.32	0.29	0.0105
	Pure error	5	56.52	11.30		

### In-vitro drug release assay of CLE

**Fig. 2:**
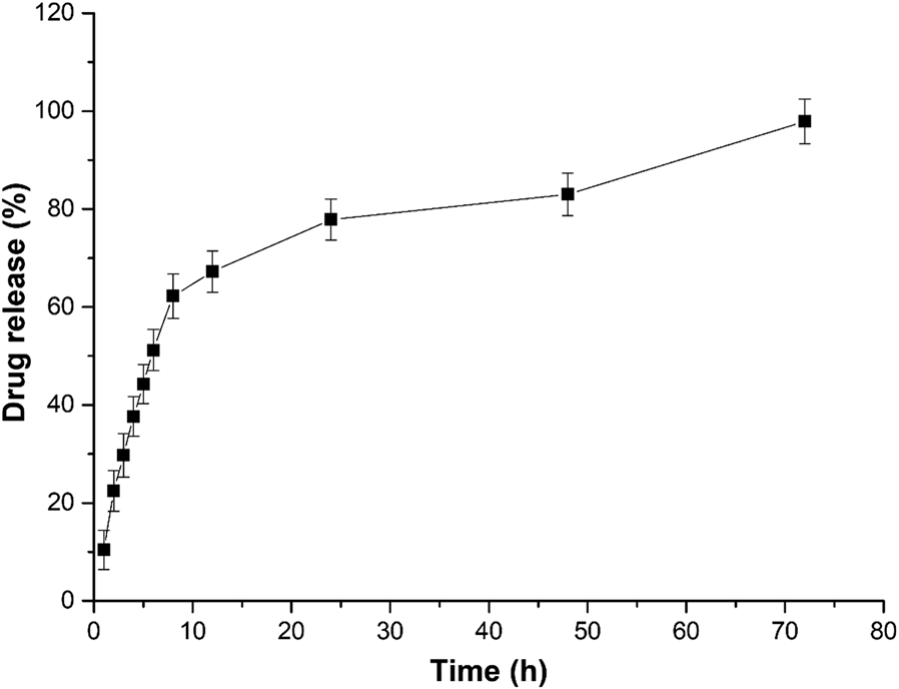
*In-vitro* drug release studies of optimized carvedilol-loaded ethosomal formulation (EF1). The experiments were carried out in triplicate and results are shown as mean ± SEM

The *in-vitro* drug release pattern obtained from the synthesized CLE formulations (EF1—EF20), showed that almost all the formulations had a linear profile release up to 8 h and then the curve plateaued (Additional file 1: Fig. S1). It was observed that the CLE prepared from EF1 formulation (CLE-EF1) had a better sustained release which lasted up to 72 h (Fig. 2), as compared to other simulated formulations. It has been known that for transdermal drug delivery the ideal size of ethosomal vesicle is < 300 nm [31, 32], suggesting that CLE-EF1 is suitable for transdermal delivery. The histogram and zeta potential of EF1 is shown in Additional file 1: Fig. S2. Therefore, CLE-EF1 was chosen for further studies.

### Physicochemical properties and In-vitro drug release assay of carvedilol-loaded ethosomal hydrogels (CLEG)

The composition of each gel formulation is shown in Table 7, and the physicochemical properties of formulated gels are shown in Table 8. The pH values of the gels were within the expected range that is suitable for skin (pH 5.5–6.8), suggesting a low possibility of skin irritation [33]. The spreadability of the gels was higher than the ethosomes. In addition, the viscosity of ethosomes-loaded hydrogels was less than the free drug-loaded hydrogel, possibly due to the presence of ethanol in the ethosomes [34]. In all the gel formulations, the drug content was found to be above 90%.

**Table 7:** Composition of carvedilol ethosomal gel formulations

Ingredients	G1	G2	G3	G4	G5	G6	G7
Carvedilol pure drug (mg)	–	–	–	–	–	–	6.25
Carvedilol-loaded EF1 (mg)	6.25	6.25	6.25	6.25	6.25	6.25	–
Carbopol-934% (w/w)	0.5	1	1.5	2	2.5	–	1

**Table 8:** Physicochemical studies of carvedilol ethosomal gel formulations

Formulation Code	Viscosity (Pa.s)	pH value	Spreadability (g.cm/sec)	Drug content (%)
G1	1.2 ± 0.2	5.44	7.80 ± 0.28	94.57 ± 0.54
G2	1.8 ± 1.0	5.68	8.24 ± 0.32	99.82 ± 0.62
G3	2.8 ± 1.5	5.81	6.57 ± 0.17	98.43 ± 0.23
G4	3.4 ± 0.52	5.54	6.20 ± 0.33	97.81 ± 0.13
G5	9.3 ± 1.0	5.93	6.82 ± 0.48	98.24 ± 0.44
G6	19.7 ± 1.5	5.84	5.37 ± 0.12	99.21 ± 0.46
G7	25.6 ± 5.7	5.61	7.90 ± 0.36	99.50 ± 0.25

**Fig. 3:**
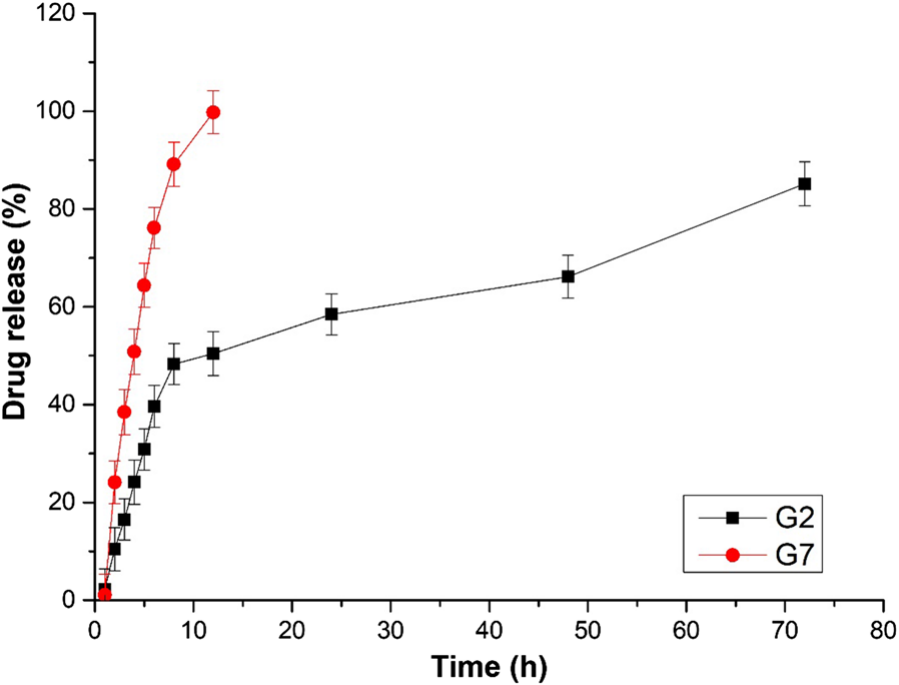
*In-vitro* drug release studies of carvedilol-loaded ethosomal hydrogel formulation (G2), and free drug-loaded hydrogels (G7). The experiments were carried out in triplicate and results are shown as mean ± SEM

The *in-vitro* drug release studies from different CLEGs (G1 – G7) are shown in Additional file 1: Fig. S3. The G7 gel showed a linear drug release, releasing > 99% of the drug within the first 12 h. The formulations (G1—G6) showed a linear release profile for the initial 8 h, releasing around 50–80% of the drug. However, in G2 < 50% drug release was observed for the initial 8 h, followed by a slow and sustained drug release pattern till 72 h (Fig. 3). It is possible that the drug release from the hydrogels depends on the presence of three-dimensional polymeric cross-links in hydrogels, which in turn is governed by an inverse relationship with the viscosity of the hydrogels [35], although this needs to be further evaluated. Hence, the G2 formulation was selected as the optimized CLEG (CLEG-G2) for further *ex-vivo* permeation studies.

### Characterization of CLE and CLEG

**Fig. 4:**
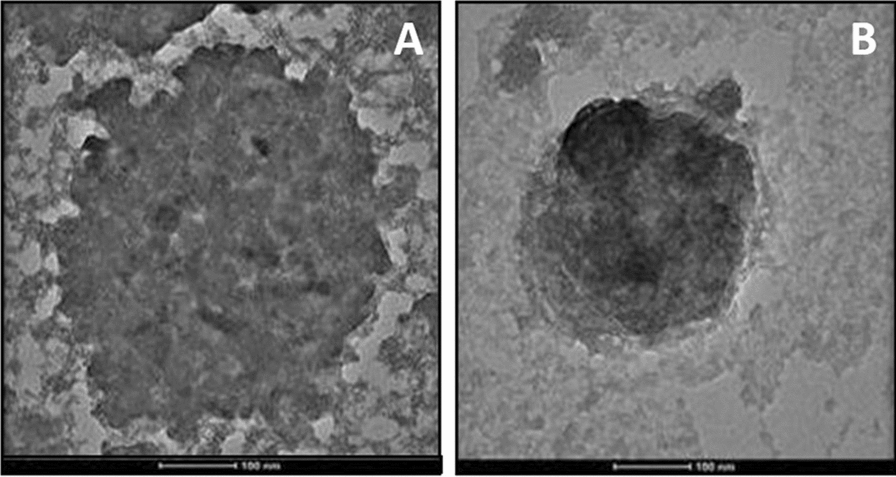
Transmission electron microscopic images of ethosomal gel at the scale of 100 nm (**a**), and ethosomes at the scale of 100 nm (**b**)

**Fig. 5:**
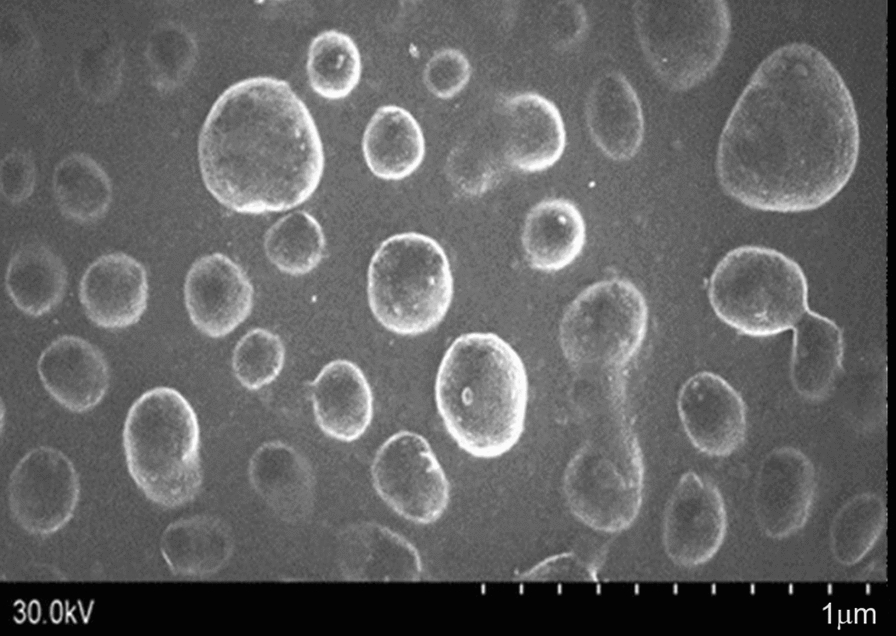
Scanning electron microscopic images of ethosomal gel at the scale of 1 m

Transmission electron microscopy (TEM) was used to examine the periphery of CLE-EF1. It was observed that the CLE-EF1 were multilamellar and smooth-surfaced (Fig. 4). The scanning electron microscopy (SEM) image of CLEG-G2 showed that the ethosomes were nearly spherical in shape and evenly dispersed in the hydrogel with minimal aggregation (Fig. 5).

**Fig. 6:**
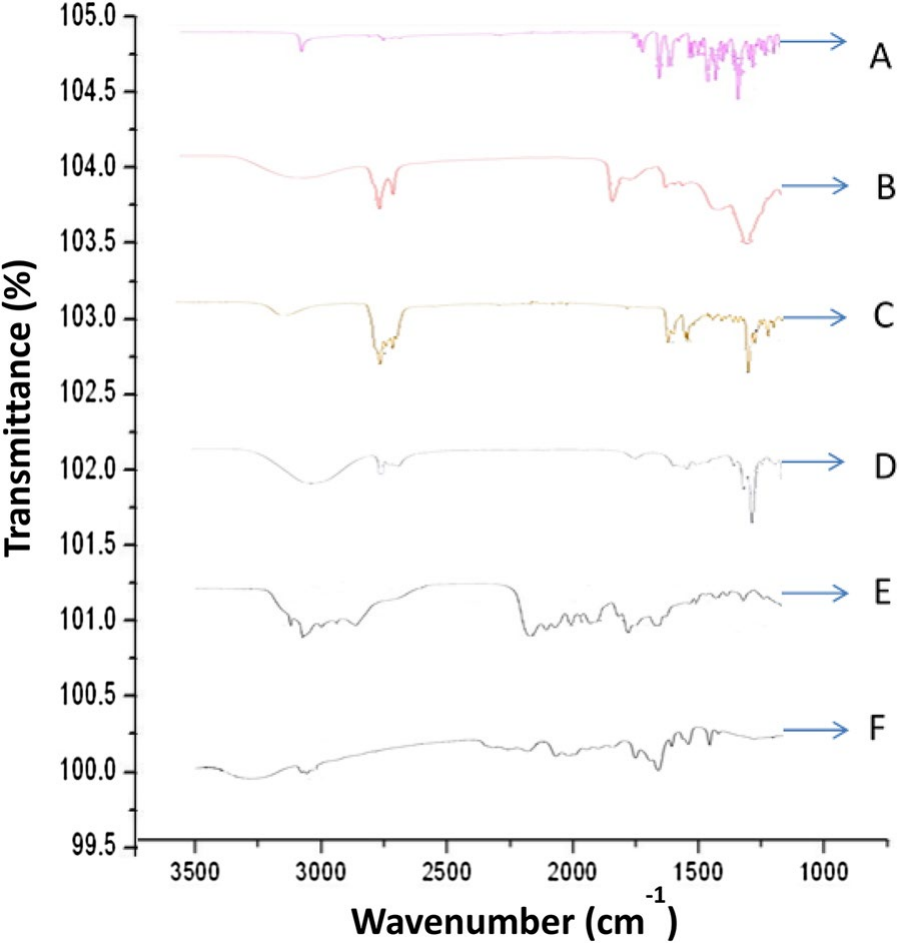
FT-IR spectrum of carvedilol (**a**), phospholipid (**b**), cholesterol (**c**), ethosomal suspension (**d**), Carbopol-934 (**e**), and ethosomal gel (**f**)

Fourier transform infrared spectroscopy (FT-IR) spectra of each component namely carvedilol, phospholipid, cholesterol, CLE-EF1, carbopol-934 and CLEG-G2 were taken. Carvedilol showed characteristic peaks of O–H and N–H stretching (3342.89 cm^−1^), C = C (1443 cm^−1^), and C-N (1347 cm^−1^ to 1251 cm^−1^) (Fig. 6). However, these peaks are either disappeared or shifted in the prepared CLE-EF1, possibly due to the entrapment of carvedilol in the ethosomes. Moreover, CLE-EF1 showed broadening in O–H stretching at around 3200 cm^−1^, indicating the formation of hydrogen bonds. In CLEG-G2, the disappearance of C-H stretching at around 2900 cm^−1^ (–CH_2_) and 3010 cm^−1^ (R-CH_2_) of carbopol-934 was observed. In addition broadening of O–H stretching at around 3400 cm^−1^ was also observed. Moreover, in CLEG-G2, there is a change in intensity and shift in the peak of carbopol-934 at around 1500 cm^−1^ (of –C–C vibration), possibly due to development of hydrogel network. The shifts in the peaks of ethosomes between 1200 cm^−1^ and 1400 cm^−1^ in the ethosomal gel indicates successful entrapment of ethosomes in the hydrogel.

### Ex-vivo skin permeation and skin retention assay

**Fig. 7:**
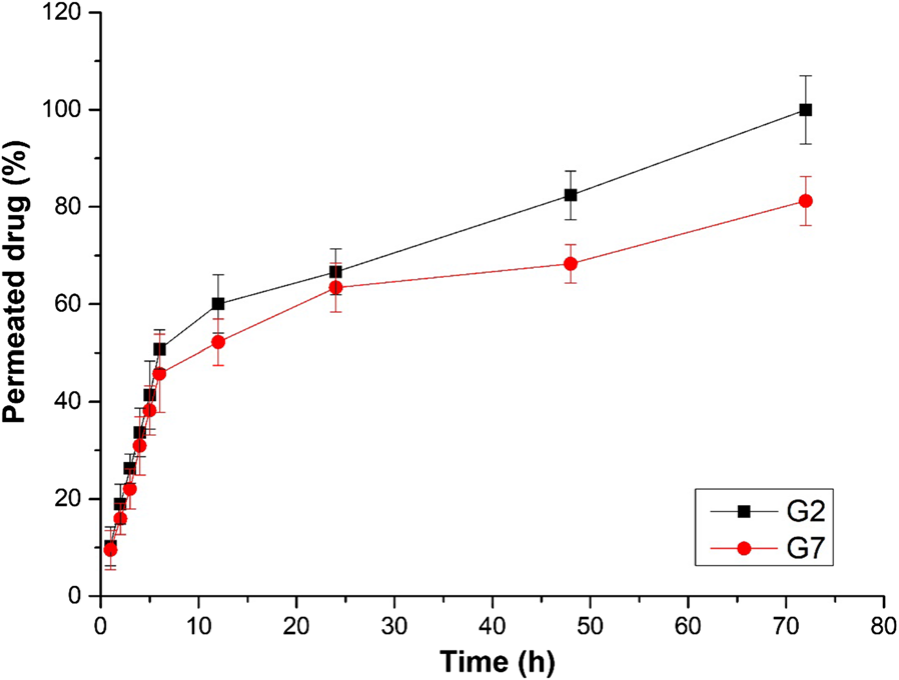
*Ex-vivo* skin permeation studies was done of on Wistar Albino rat skin for carvedilol-loaded ethosomal hydrogel formulation (G2), and free drug-loaded hydrogels (G7). Data are represented as the mean ± SEM of six mice

The *ex-vivo* permeation studies were performed for CLEG-G2, where G7 was taken as control (Fig. 7). It was observed that the CLEG-G2 showed considerably higher amounts of drug permeated through the skin with higher steady-state flux (Jss) (89.64 ± 7.26 µg.cm^−2^.h^−1^), as compared to the control G7 hydrogel (54.59 ± 6.21 µg.cm^−2^.h^−1^), possibly due to increased drug release from the hydrogel (Fig. 3), and low viscosity (Table 8). Moreover, the skin retention studies of the CLEG-G2 also showed better retention capacity (10.86% ± 3.21), compared to G7 (4.63% ± 1.23).

### In-vivo anti-hypertensive study

**Fig. 8:**
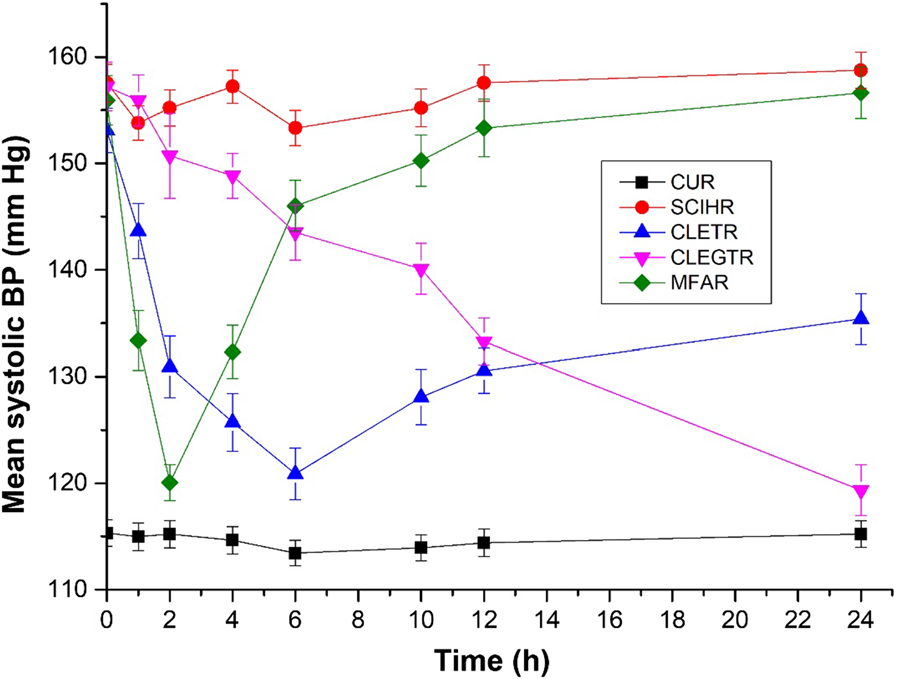
Anti-hypertensive effect of carvedilol-loaded ethosomes and carvedilol-loaded ethosomal gels in sodium chloride induced hypertensive rat model. *CUR* Control untreated rats, *SCIHR* Sodium chloride induced hypertensive rats, *CLETR* carvedilol-loaded ethosomes treated rats, *CLEGTR* carvedilol-loaded ethosomal gel treated rats, *MFAR* Marketed formulation administered rats. Data are represented as the mean ± SEM of six mice

**Fig. 9:**
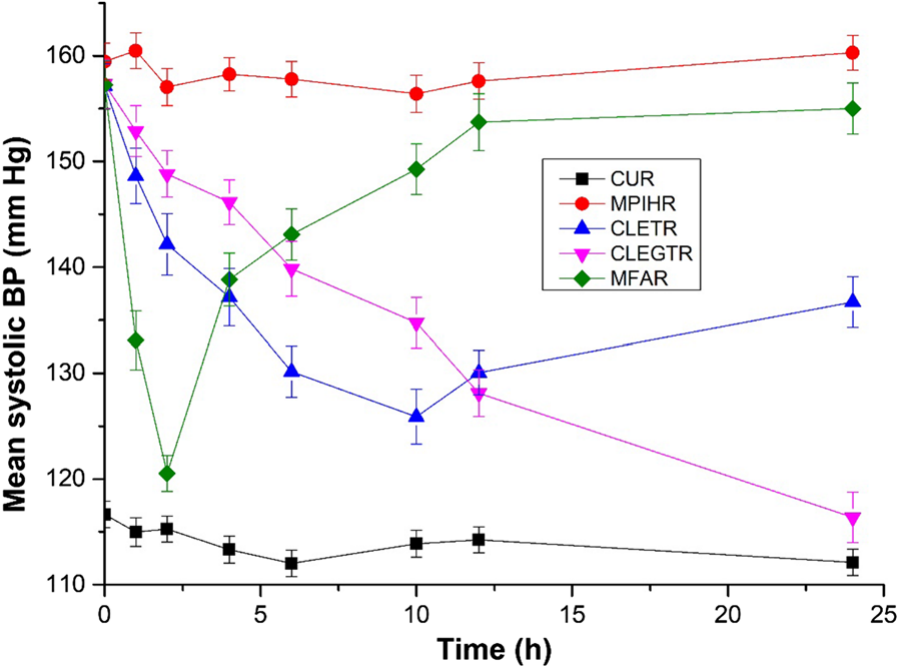
Anti-hypertensive effect of carvedilol-loaded ethosomes and carvedilol-loaded ethosomal gels in methyl prednisolone induced hypertensive rat model. *CUR *Control untreated rats, *MPIHR *Methyl prednisolone induced hypertensive rats, *CLETR *carvedilol-loaded ethosomes treated rats, *CLEGTR* carvedilol-loaded ethosomal gel treated rats, *MFAR* Marketed formulation administered rats. Data are represented as the mean ± SEM of six mice

From Figs. 8 and 9, it can be observed that in the control untreated rats (CUR) the systolic blood pressure (BP) remained normal throughout the study. The oral administration of marketed carvedilol formulation exhibited a rapid decrease in the systolic BP and normalizing it within 10 h. Meanwhile, in the case of both CLE-EF1-treated rats (CLETR), and the CLEG-G2-treated rats (CLEGTR), a gradual slow reduction in systolic BP was observed which was brought down to normal in 24 h. This exhibited sustained and extended action of carvedilol in both CLETR and the CLEGTR is possibly due to the slow release of carvedilol.

## Discussion

Transdermal application of ethosomal hydrogels is the well-known mode of delivery for lipophilic drugs. The present study aimed to optimize and develop carvedilol-loaded ethosomal hydrogel for transdermal delivery to overcome the limitations associated with oral administration of carvedilol. Here, RSM studies showed that the ethosomes formation strongly depends on the amount of phospholipid, ethanol and PG used in the formulation that might influence the vesicle size, % EE and the % CDR.

It is well-known that the ethosomal vesicle size is an important factor for effective transdermal delivery of payload [31]. High ethanol concentration is generally suggested to reduce the size of ethosomes [17]. However, excess of ethanol is also not recommended as it might disrupt the self-assembly of lipid molecules, increasing the size of ethosomes due to disintegration of the structural integrity of the ethosomes [36, 37]. Moreover, ethanol also possesses a fluidizing effect on the phospholipid bilayer [19]. The formation of ethosomal vesicles also depends on the diffusion rate of ethanol into the water phase, impelling the precipitation of phospholipids which might affect the size of the vesicles formed. Therefore, optimal ethanol concentration is important to prepare highly stable ethosomes [38]. Moreover, there is a correlation between ethanol concentration and other constituents used in the synthesis of ethosomes. It was also observed that at constant ethanol concentrations, the increase in phospholipid concentration decrease the vesicle size. This is possibly due to the ability of phospholipid to enhance the rigidity of the vesicles, owing to the high concentration of phospholipid macromolecular chains.

A high level of % EE is always preferred because it helps in transporting sufficient drugs to the site of action [39]. The increase in the % EE due to an increase in ethanol concentration can be attributed to an increase in the membrane fluidity [40]. The increase in ethanol concentration improves the solubility of the hydrophobic drug carvedilol in the inner polar ethosomal core which also causes an increase in % EE. However, very high ethanol concentration can solubilize the phospholipids present in the ethosomal membrane and can cause destabilization of the ethosomal membrane making it leaky and hence decreasing the % EE [41]. The addition of phospholipid can increase the number of vesicular bilayers formed, thereby increasing the drug holding capacity of the ethosomes, hence increasing % EE [37]. Since the encapsulated drug carvedilol is lipophilic, the phospholipid molecules could easily entrap the drug. The hydrophobic nature of carvedilol also ensure very low drug loss into the surrounding aqueous phase during the formulation of ethosomes [42].

The high concentration of ethanol in ethosomes can cause steric stabilization of the vesicles by imparting a net negative charge on the ethosomal surface [43]. The ethosomes are prevented from aggregation due to electrostatic repulsion, rendering stability to the ethosomes [43]. The ethanol molecules can distribute both within the outer lipid bilayers and in the inner aqueous region of the vesicles [44]. The hydrophilic terminal hydroxyl groups of ethanol molecules that gets distributed within the vesicular lipid bilayers can reach out to interact with the outer hydrophilic phase, leading to the formation of hydrogen bond [44]. The hydroxyl groups of the ethanol molecules can also form hydrogen bond among themselves, imparting a net negative charge to ethosomes. Hence, the zeta potential value becomes more negative [45].

The biphasic release pattern was observed in an *in-vitro* drug release assay. The burst release at initial time points is possibly due to the drug particles associated with the vesicle surface, followed by the slow release of the drug entrapped in the ethosomal core [12]. It is plausible that the high % of ethanol (~ 40% (v/v)) can increase the drug release at early time points due to increased fluidity of the ethosomal vesicle and increased drug solubility in the hydro-ethanolic core of ethosomes [46]. Moreover, significantly high drug release at early time points when high % (v/v) of PG is used is possibly due to increase in the permeability and the wet ability of the vesicles [47]. The multilamellar nature of the formulated ethosomes is possibly due to the presence of ethanol, which might contribute to the flexibility and fluidity of the phospholipids bilayers [38].

Previous pharmacokinetic studies have shown that the transdermal delivery of carvedilol has provided steady-state carvedilol level in blood plasma with improved bioavailability [11, 48]. The ethosomal suspension-loaded hydrogels had better permeation capability than the free drug-loaded hydrogel because of the influential role played by ethanol in fluidizing the lipids present in both the vesicles and in the SC, hence providing better malleability for the ethosomes [49]. The presence of phospholipids also helped in providing a better penetrative effect for the ethosomes by ensuring an effective mixing of the vesicles with the skin lipids thereby leading to the opening of SC [50]. In addition, it is also plausible that the higher drug permeation across the skin from ethosomal hydrogel is due to its low viscosity, as compared to the free drug-loaded hydrogel. It has been recognized that there is an inverse relationship between drug permeation and the viscosity of hydrogel [51]. However, very low viscosity is also not advisable as it can reduce the contact time between the skin and the hydrogel [52]. Hence optimum viscosity of hydrogel is essential for better drug permeation.

In skin retention studies, the ethosomal gel exhibited a better retention effect than the control hydrogel. The presence of high concentration of ethanol in ethosomes allowed better carvedilol penetration through the skin. This is because ethanol can disturb the SC organization by fluidizing the skin lipids ensuring deeper drug penetration through the skin layers [37]. Ethanol also imparted flexibility to the ethosomes which allowed them to pass easily through the skin pores having smaller diameters compared to ethosomes [41]. Moreover, the phospholipid content in ethosomes may assist in the retention of carvedilol for a longer time due to the fusion of the ethosomes with the skin lipids [53].

## Conclusions

Taken together, the use of Central composite design-mediated optimization of ethosome formulations can help in better understanding of the correlation among the variables involved in ethosome formation and their effects on vesicle size, entrapment efficiency, and cumulative drug release. It was observed that synthesized CLEs exhibit low vesicle size, high entrapment efficiency, and high cumulative drug release. The ethosomes-loaded hydrogel showed a controlled release of carvedilol with higher skin permeation and skin retention of carvedilol compared to free drug-loaded hydrogel. These as-synthesized ethosomal hydrogels also showed a better anti-hypertensive effect. This study suggests that carvedilol-loaded ethosomal hydrogels might be an effective dermal delivery system for the controlled delivery of carvedilol against hypertension in the future.

## Materials and methods

### Materials

The drug, carvedilol, was procured from Chandra Laboratories, Hyderabad India. Soy lecithin, ethanol, PG (mol. wt. 76.09 g/mol), tri-ethanolamine and carbopol-934 were purchased from Research Lab Fine Chem Industries, Mumbai, India while cholesterol was obtained from Merck Ltd., Mumbai, India. The purchase of ultrapure water was done from Cortex Laboratories, Hyderabad, India. Centrisart filters with molecular cut off at 20,000 were purchased from Sartorius Research Lab Fine Chem. Industries, Mumbai, India. Transcutol P was obtained from Triveni Chemicals, Gujarat, India**.** The remaining chemicals used were of analytical grade and solvents were of HPLC grade.

### Solubility studies of carvedilol

The solubility of carvedilol was tested using ethanol and distilled water solutions at varying volumetric ratios, i.e. 20:80, 30:70, 40:60 and 50:50% (v/v) with an excess of the drug in 1.5 ml of each vehicle in separate micro centrifuge tubes. After vortexing, the centrifuged tubes were kept for incubation in an orbital shaker (Remi Electrotechnik Ltd, Mumbai, India) for 48 h at an ambient temperature of 25 °C to ensure proper solubilization [54, 55]. For removal of the excess undissolved drug, the incubated samples were centrifuged at 3000 rpm. The supernatant taken at regular intervals were quantified for determining the drug concentration using reversed-phase high-performance liquid chromatography (RP-HPLC) method. The experiments were carried out in triplicate and results are shown as mean ± SEM.

### HPLC analysis

The high-performance liquid chromatography (HPLC) system used was Shimadzu LC–10AT with SPD–10A UV/Vis as a detector and LC10 software for carvedilol quantification. The column used was Kromasil RPCL C18 short column (150 × 6 mm, 5 µm). The mobile phase composition was acetonitrile and 15 mM ortho-phosphoric acid in the volumetric ratio of 37:63 (v/v) and triethylamine was added to the solution at the concentration of 0.25% (v/v). The pH value of the mobile phase was adjusted to 2.5 using ortho-phosphoric acid. 20 µl of each sample was injected into the rheodyne injection port. The flow rate was 1 ml/min with a runtime of 12 min. The retention time for carvedilol was 4.2 min. The exiting eluent was monitored at a wavelength of 242 nm [56].

### Optimizing of ethosomal formulation

The CCD model was implemented to predict the optimized formula of the preparation of CLE. The design of experiments (DoE) software (Version 11, Stat-Ease Inc., Minneapolis, USA) was used for the RSM study. For CCD modelling, the variables are chosen to be either dependable or independent. The independent variables were the different constituents of ethosomes, namely the amount of phospholipids (X_1_) % (w/v), amount of ethanol (X_2_) % (v/v) and amount of PG (X_3_) % (w/v), keeping amount of carvedilol (6.25 mg) and cholesterol (0.0005% (w/v)) constant. While the dependent variables (i.e., responses) were the vesicle size (Y_1_), % EE (Y_2_), and % CDR (Y_3_). Based on the experimental setup and number of factors involved in the formulation, a quadratic relation between the factors was chosen governed given by Eq. 4. The ANOVA method was used to know the significant effect of the factors and their interactions.4where, X_1_, X_2_ and X_3_ are the independent variables % Phospholipid, % Ethanol and % PG, respectively. Y is the dependent variable. B_i_ and B_ij_ are the coefficients associated with each variable. € is the experimental error.

### Preparation of carvedilol loaded ethosomes

The ethosomes were prepared by a cold method using ethanol [(20–40% (v/v)], PG [5–10% (v/v)], 2–5% (v/v) soya phospholipids and 0.005% (w/v) cholesterol. The soya phospholipids, PG, cholesterol and carvedilol (6.25 mg) were added to ethanol gradually followed by vigorous stirring. The mixture was heated to 30 °C in a water bath and distilled water (10 ml) was added slowly drop wise whil0e the mixture was being stirred magnetically at 700 rpm for 15 min. After the addition of water, the stirring was carried out for an additional 5 min. The formed ethosomal suspension was then sonicated for 5 min to reduce the vesicular size [57]. The final step was refrigeration of the suspension at 4 ℃ [58].

### Preparation of carvedilol loaded ethosomal hydrogel

The hydrogel was formulated using various concentrations of the polymer carbopol 934, i.e. 0.5% (w/w), 1% (w/w), 1.5% (w/w), 2% (w/w) and 2.5% (w/w). Accurately weighed quantities of the polymer were dissolved in specific quantities of the prepared ethosomal suspension or free drug (Table 7), using a magnetic stirrer at 1000 rpm. The process was continued until smooth lump-free homogenous gels were attained. An appropriate quantity of tri-ethanol amine was added to adjust the pH to 5.5 during gel preparation. The final semi-solid gel was stored overnight at room temperature.

### Characterization of ethosomes/ethosomal gel

#### Assay of encapsulated drug

The amount of encapsulated drug in CLEs and CLEGs were calculated using HPLC. The diluents used for dissolving the prepared CLEs and CLEGs were chloroform and methanol in 1:1 (v/v) ratio. The solution was then centrifuged in centrisart tubes at 8000 rpm for 30 min. The free unencapsulated drug concentration present in the supernatant was determined by HPLC and % EE was calculated using Eq. 5 [12].

5 $$\%{EE}= \left[\frac{\begin{array}{cc}{A}_{total}- {A}_{unentrapped}\end{array}}{{A}_{total}}\right]\times 100.$$

where, A_total_ = total amount of carvedilol; A_unentrappe**d**_ = unentrapped carvedilol.

#### In-vitro drug release studies

*In-vitro* drug release assay was performed for CLSs (EF1–EF20), and CLEGs (G1–G7). The dialysis bag method was used to carry out the *in-vitro* release studies. Before the test, it was made sure that the membrane of the dialysis was properly hydrated with complete wetting of the membrane [59]. The hydration medium used was phosphate buffer solution (PBS) of pH 6.8 and the hydration was carried out for 2 h. The samples were transferred to the dialysis bags with both ends sealed. The bags were then suspended in bottles containing 200 ml of the buffer solution and rotated at 100 rpm in a thermostatically temperature-controlled water bath shaker. The temperature was maintained at 37 ± 0.5 °C throughout the process. For each sample, 1 ml of the aliquot was taken at pre-determined time intervals. The aliquot was taken on an hourly basis for the first 6 h and then after the sample was taken after 8, 12, 24, 48, and 72 h. The drug concentration after each time interval, was determined at 242 nm using HPLC as previously mentioned. The experiments were carried out in triplicate and results are shown as mean ± SEM.

#### Dynamic light scattering (DLS) studies

The average vesicle size, PDI, and zeta potential were determined by using dynamic light scattering zeta sizer (Malvern Nano-ZS90). To avoid the error due to multi-scattering action, a 2 ml quantity of each sample was undergone dilution with distilled water by proper mixing. The diluted sample was then injected into a clean disposable zeta cell and measurements were recorded.

#### Vesicle size and morphology studies

The shape and size of the prepared CLE-EF1 and CLEG-G2 were observed using TEM. The sample preparation was done by placing a drop of the diluted ethosomal suspension on a carbon-coated grid and followed by the addition of a drop of aqueous 2% phosphotungstic acid solution. After the removal of excess liquid, the suspension was air-dried and TEM imaging was done at an acceleration voltage of 100 kV [60]. SEM was used to examine the surface morphology of the prepared ethosomes. After adhering the ethosomal suspension onto the carbon-coated stubs, they were sputtered with platinum using a coating machine (Auto Fine Coater, JFC-1600, JEOL, Japan) and then observed under the SEM (JSM-6501LA, JEOL, Japan), in a high vacuum atmosphere at 12 kV and 30.0 kV [61, 62].

#### FT-IR studies

The K-Br pellet technique was used for FT-IR studies. The scanning range and the resolution were kept at 400–4000 cm^−1^ and 4 cm^−1^, respectively [63]. The FT-IR instrument used was of making Bruker Optics Germany Model-200.

#### Physical examination and pH measurement of ethosomal gel

The physical characteristics of the prepared hydrogels were determined by visual examination. The gel samples were visually examined to determine the homogeneity, consistency, phase separation and appearance of any aggregate formations. The pH was measured by using a digital pH meter (Remi, Hyderabad, India). For proper measurement, it was ensured that the glass electrode of the pH meter was completely dipped into the gel system [18].

#### Viscosity measurement of gels

The viscosity was measured using a viscometer (Brookfield Viscometer, CAP 2000L), equipped with cone spindle number 1. The analysis was done under high torque and low-temperature mode. About 500 mg of each sample was taken for analysis. 5 min of prior settling time was ensured before viscosity determination [33].

#### Spreadability of the gels

The degree of gel spreadability was measured using the glass slide apparatus with the help of a modified wooden block. Using a glass side, a quantity of gel of known weight was placed on the movable pan and then placed on the fixed glass slide to make sure the gel was properly sandwiched between the glass slides for 5 min duration. 500 g weight was placed on the glass plate till maximum spreading. The excess gel exiting from the sides was continuously removed. The spreadability was determined using Eq. 6.

6 $$S = M/T.$$

where, S = spreadability (g.cm/sec) M = mass (g), T = time (sec).

### Animals

All the animal studies were conducted on Wistar Albino Rats after obtaining permission from the Committee for the purpose of control and supervision of experiments on animals (CPCSEA) with the wide permission being documented as No.51/01/C/CPCSEA/2013/13. Wistar Albino Rats (200–250 g) were fed ad libitum as per the standard procedure. All mice were maintained on a light/dark cycle (12/12-h), at 21–25 °C temperature and 40–60% humidity. The rats were allowed to adapt for 14 days before the experiment.

### Ex-vivo skin permeation studies

The *ex-vivo* skin permeation studies was carried for CLEG-G2, and was compared with pure drug-loaded control gel (G7). After sacrificing the rats (n = 6 for each group), the skin from the abdominal portion was chosen for conducting the studies. The hair from the skin was removed thoroughly using a razor blade and the skin was separated from the connective tissue diligently using a scalpel to prevent perforations or incisions. After removal, the skin was then washed thoroughly with double distilled water and stored at − 18 °C to retain its metabolic efficiency. The skin was then hydrated overnight at 25 °C in PBS (pH 6.8 and containing 0.02% sodium azide as a preservative). The overnight hydration was done to ensure the removal of extraneous debris and leachable enzymes [64, 65].

A skin sample of appropriate size was fixed at the ends of a diffusion cell ensuring a permeation area of about 5.3 cm^2^ was available. The SC portion of the skin layer faced the donor compartment while the dermis side of the skin met the receptor compartment. A 200 ml of a solution of transcutol, ethanol and PBS at pH 6.8 in the ratio of 10:40:50 (v/v) was used as the hydration medium [32]. The diffusion cells were maintained in a thermostatically controlled water bath shaker at 37 ± 1 °C at 100 rpm. At pre-determined time intervals (0, 1, 2, 3, 4, 5, 6, 12, 24, 48 and 72 h), a 5 ml sample of the receptor medium was withdrawn and again filled with the same amount of hydration medium to ensure proper sink condition. The withdrawal samples were filtered using a nylon syringe filter of 0.22 μm size. Every time a sample was taken, an equivalent amount of fresh receptor medium was added to maintain the volume constant. The assay of the drug in the sample taken was determined at 242 nm using HPLC. A graph was plotted for the cumulative drug permeation through the skin against time to see the release pattern. The steady-state flux (J_ss)_ was calculated from the slope of the linear portion of the drug permeation study graph and it can be expressed as Eq. 7 [66].

7 $${\mathrm{J}}_{\mathrm{ss}}={\mathrm{DK}}_{\mathrm{m}}{\mathrm{C}}_{\mathrm{v}}/\mathrm{h}$$

where, K_m_ = partition coefficient of the drug between membrane and vehicle, D = Effective diffusion coefficient, h = thickness of the stratum corneum, C_v_ = concentration of the vehicle.

#### Skin retention studies

The depot action of the tested formulation was investigated at the end of *ex-vivo* skin permeation studies. The skin was cleaned several times using a cotton swab with methanol to remove the excess drug existing on the surface. The cleaned skin was then soaked into a 10 ml of methanol and homogenized for 5 min for extracting the drug. The samples were centrifuged at 6000 rpm for 30 min and filtered using 0.22 µm nylon syringe filter. The filtrate obtained was assayed for the drug concentration using HPLC. The measurements were done in triplicate and compared with those of carvedilol control gel formulation.

#### Anti-hypertensive study

Two different methods (based on hypertensive inducing models), were used for evaluating the anti-hypertensive effect of ethosomes (CLE-EF1) and its gel formulation (CLEG-G2), in comparison with marketed formulation of carvedilol. The hypertensive effect was induced by either sodium chloride (SCIHR group of rats) or methyl prednisolone (MPIHR group of rats). After two weeks from inducing hypertensive effect, the rats (n = 6 for each group) in which the mean systolic BP was 150–160 mm Hg were selected and the drugs were administered. Both the SCIHR and MPIHR groups, were treated transdermally with CLE-EF1 (CLETR group of rats, 10 mg/kg of body weight) and CLEG-G2 (CLEGTR group of rats, 10 mg/kg of body weight). The marketed formulation carvedilol drug (MFAR group of rats) was administered orally (10 mg/kg of body weight, tablets USP). The dose is selected based on the previous studies [67]. In both the groups, control untreated rats (CUR) were taken as control and were not administered with any hypertensive inducing agent or any treatment. Before the BP measurement was done, the rats were properly trained to stay calm and non-aggressive in the cages. The systolic BP was measured by the tail-cuff method (Bio-pack system Inc., Santa Barbara, USA) at pre-determined time intervals (0, 1, 2, 4, 6, 10, 12, and 24 h), after the drug administration for all the groups [68, 69].

## Supplementary Information

**Additional file 1: Fig. S1.** In-vitro drug release profile for ethosomal formulations EF1-EF20. **Fig. S2** A) Histogram showing the size distribution of ethosomal formulation EF1 B) Zeta potential of EF1. **Fig. S3** In-vitro drug release profile of ethosomal gels G1-G7

## Data Availability

All data and materials used are all available in the manuscript.
