# Correction to: Central composite design for the development of carvedilol-loaded transdermal ethosomal hydrogel for extended and enhanced anti-hypertensive effect

**DOI:** 10.1186/s12951-021-00944-y

**Published:** 2021-07-19

**Authors:** Padmanabha Rao Amarachinta, Garima Sharma, Noufel Samed, Ananda Kumar Chettupalli, Madhusudhan Alle, Jin-Chul Kim

**Affiliations:** 1 School of Pharmacy, Anurag University, Telangana, 500088 India; 2 Department of Biomedical Science & Institute of Bioscience and Biotechnology, Kangwon National University, Chuncheon, 24341 Republic of Korea

## Correction to: J Nanobiotechnol (2021) 19:100 https://doi.org/10.1186/s12951-021-00833-4

Following publication of the original article [1], the authors identified a change in the ‘Funding’ section. The complete ‘Funding’ section is given below

“This research was supported by Technology Innovation Program (20009663) funded by the Ministry of Trade, Industry & Energy (MOTIE, Korea). This research was also supported by Basic Science Research Program through the National Research Foundation of Korea (NRF) funded by the Ministry of Education (No. 2018R1A6A1A03025582).” The original article has been revised.
