# Supplementary material for: Central composite design for the development of carvedilol-loaded transdermal ethosomal hydrogel for extended and enhanced anti-hypertensive effect
Source: J Nanobiotechnology. 2021 Apr 9;19:100. doi: 10.1186/s12951-021-00833-4 (PMC8035747; doi:10.1186/s12951-021-00833-4)
Supplement: Supplementary file 1 — Additional file 1: Fig. S1. In-vitro drug release profile for ethosomal formulations EF1-EF20. Fig. S2 A) Histogram showing the size distribution of ethosomal formulation EF1 B) Zeta potential of EF1. Fig. S3 In-vitro drug release profile of ethosomal gels G1-G7 [file 12951_2021_833_MOESM1_ESM.doc]

*Supporting information for*

**Central composite design for the development of carvedilol-loaded transdermal ethosomal hydrogel for extended and enhanced anti-hypertensive effect**

Padmanabha Rao A^1ǂ^, Garima Sharma^2ǂ^, Noufel Samed^2ǂ^, Ananda Kumar Ch^1^, Madhusudhan Alle ^2^**, Jin-Chul Kim^2^*

^1^School of Pharmacy, Anurag University, Telangana, 500088, India

^2^Department of Biomedical Science & Institute of Bioscience and Biotechnology, Kangwon National University, Chuncheon, 24341, Republic of Korea

* Corresponding Author: Jin-Chul Kim;
Department of Biomedical Science & Institute of Bioscience and Biotechnology, Kangwon National University, Chuncheon, 24341, Republic of Korea

Tel.: +82-33 250 6561
Fax: +82 33 253 6560.
E-mail address: jinkim@kangwon.ac.kr (J.-C. Kim)

** Co-corresponding author: Alle Madhusudhan
E-mail address: [allemadhusudhan@kangwon.ac.kr](mailto:allemadhusudhan@kangwon.ac.kr)

^ǂ^These authors contributed equally

**1. Supplementary materials and methods**

***1.1 Skin irritation studies***

All the animal studies were conducted on Wistar Albino Rats rats after obtaining permission from CPCSEA with the wide permission being documented as No.51/01/C/CPCSEA/2013/13. Using a clipper, the hair from the dorsal portion of nine rats was removed and the ethosomal gel was applied on the blank skin portion. Before the application, the rats were divided into 3 groups with each group consisting of 3 members. Each group had a characteristic based on the application of the gel as follows: Group 1 - No application of gel on the rats, Group 2 -

The prepared ethosomal gel was applied on the rats, Group 3 – Blank (without drug) ethosomal gel was applied on the rats. Each time the amount taken for the application was 500 mg with uniform spreading over the blank skin portion of area 4 cm^2^. Any sign of erythema or redness of skin was observed after every 24 h up to 72 h. The time is measured from the point of gel application.

***1.2 Stability studies***

The stability studies were conducted for both ethosomal suspension and ethosomal gel. Two batches were used for each of the formulations, one was stored at 4°C and the other at room temperature at 23-30 °C. The parameters determined for stability studies were mean vesicle size, PDI, zeta potential, % EE and assay using HPLC. The stability studies were conducted at 0, 1, 2, 3 and 6 months [1,2].

**2. Supplementary results and discussion**

***2.1 Skin irritation studies***

No skin irritation, as indicated by absence of any sign of erythema or redness of skin, was observed after every 24 h up to 72 h in the mice upon gel application, possibly due to the normal pH range of formulated gels (5.5-6.8). The skin irritation test studies revealed that the ethosomes were safe to use. It is identified that carbopol has no reported skin irritation and stability issue with lipid vesicles [3].

***2.2 Stability studies***

The stability studies for both ethosomal suspensions and ethosomal gel revealed that the properties taken in consideration like the physical appearance, rheological properties, % EE all remained intact for 3 months. The stability studies were conducted at both refrigeration and room temperatures (4 ± 2 °C & 27 ± 2 °C respectively). The change in the % EE, size, PDI, zeta potential and viscosity were negligible (data not given).

**Supplementary Figures**


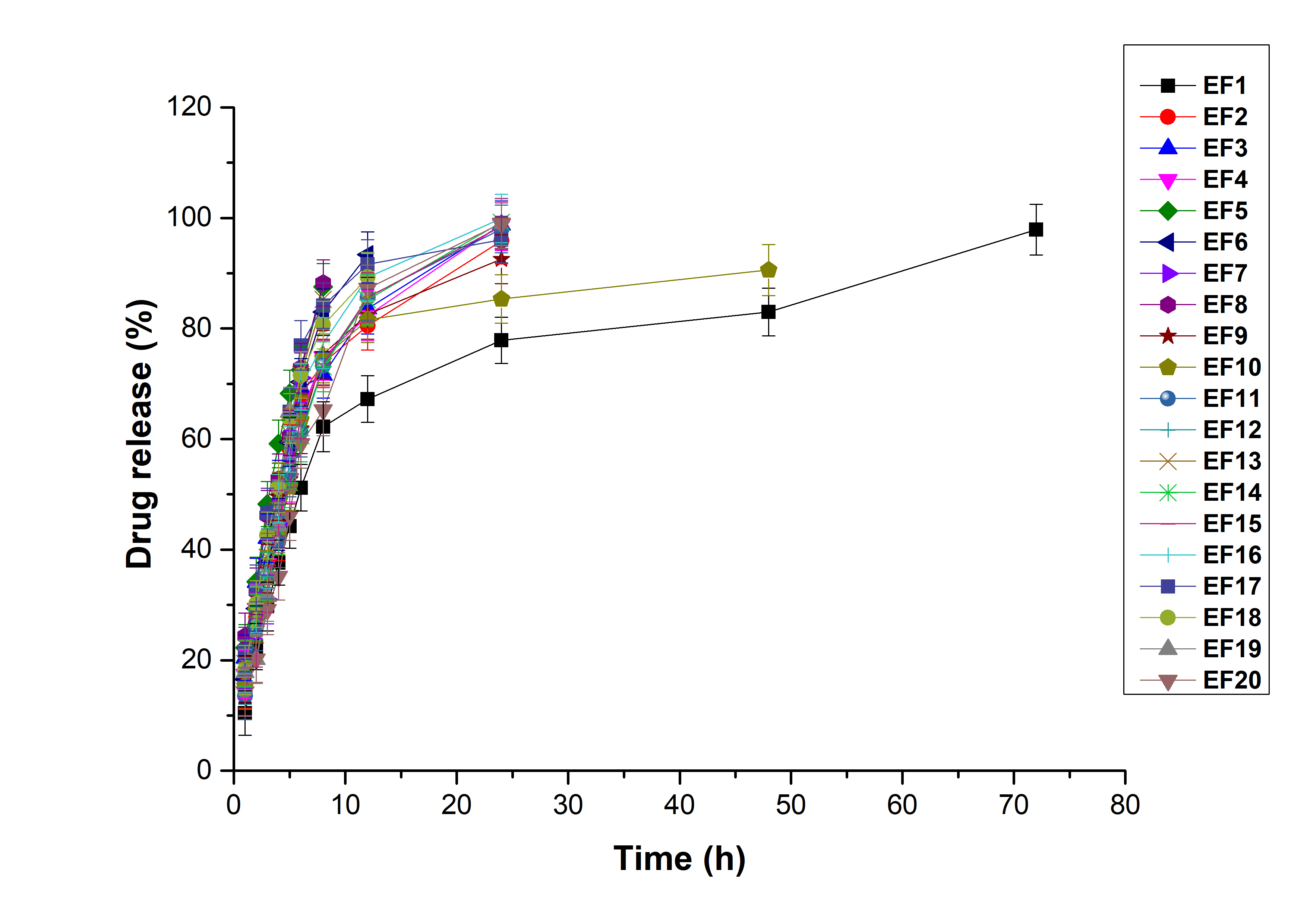


**Fig. S1** *In-vitro* drug release profile for ethosomal formulations EF1-EF20


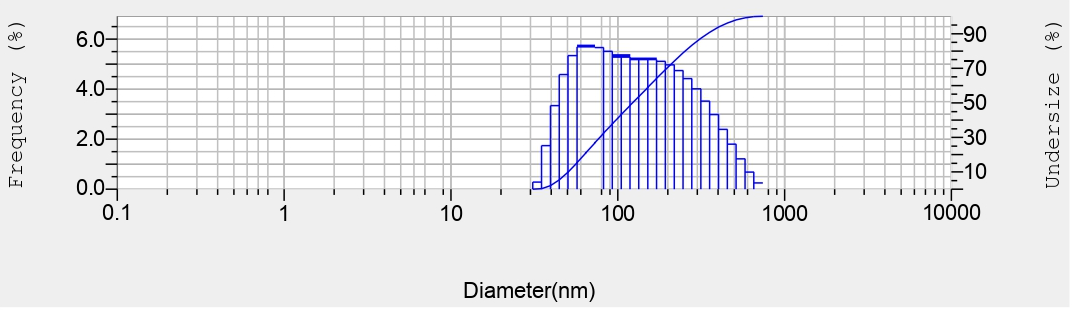


**A**

**B**


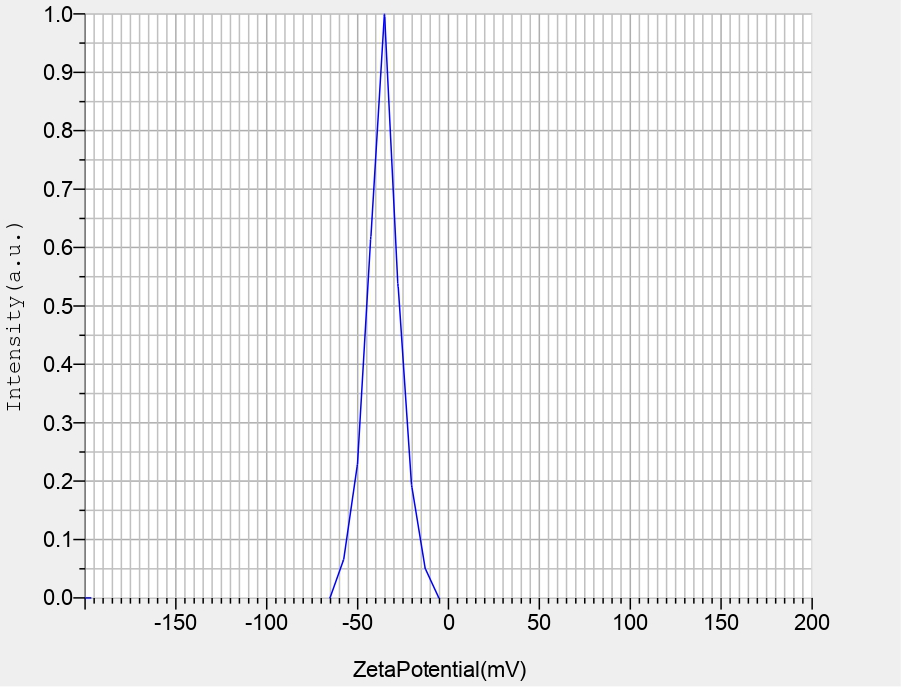


**Fig. S2** A) Histogram showing the size distribution of ethosomal formulation EF1 B) Zeta potential of EF1


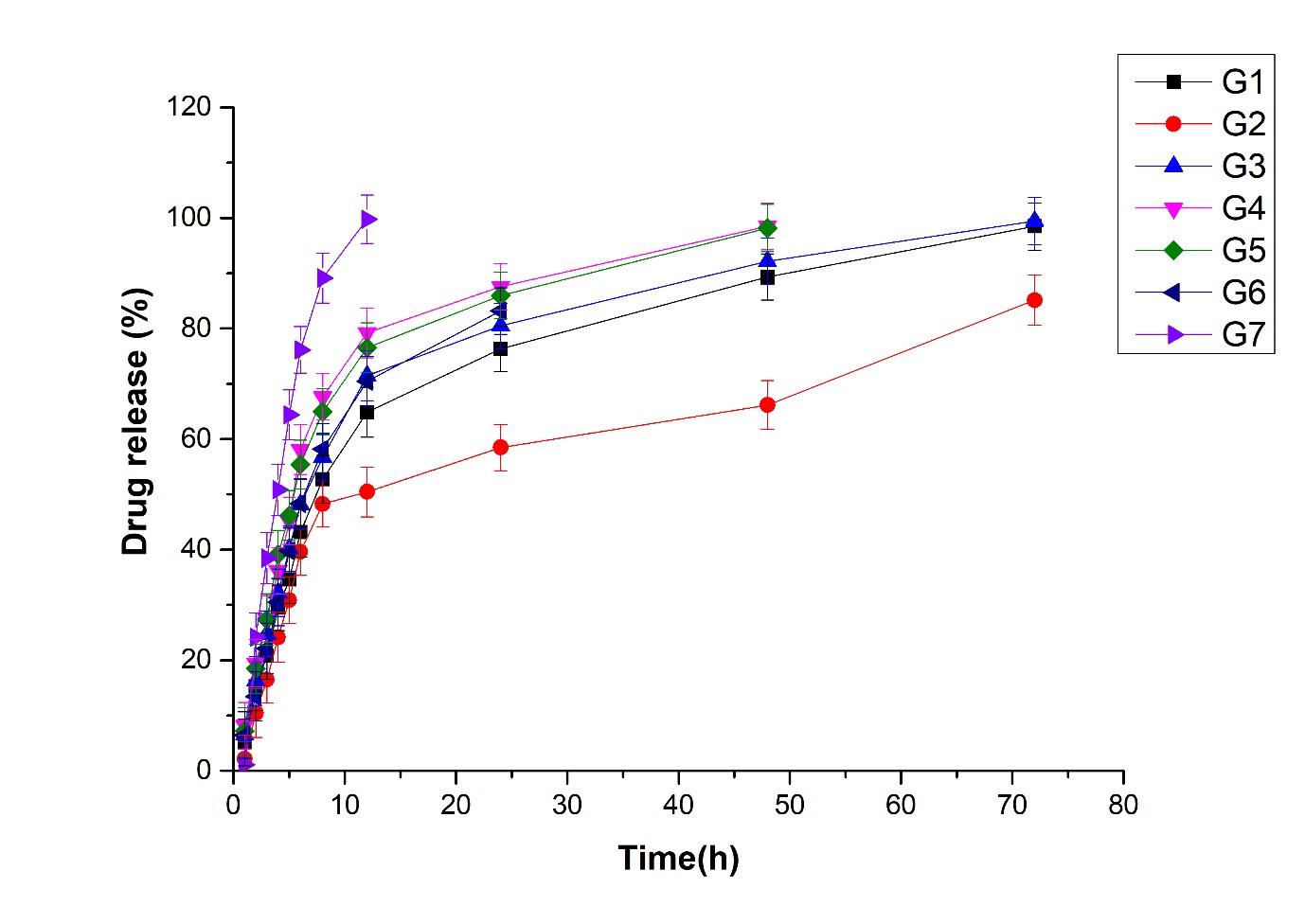

**Fig. S3** *In-vitro* drug release profile of ethosomal gels G1-G7

**References**

1. Wo Y, Zhang Z, Zhang Y, Zhang Z, Wang K, Mao X, et al. Enhanced in Vivo delivery of 5-fluorouracil by ethosomal gels in rabbit ear hypertrophic scar model. Int J Mol Sci MDPI AG; 2014;15:22786–800.

2. Zandi G, Lotfipour F, Ghanbarzadeh S, Medghalchi M, Hamishehkar H. A comparative study on the potentials of nanoliposomes and nanoethosomes for Fluconazole delivery. J Drug Deliv Sci Technol. Editions de Sante; 2018;44:264–269.

3. Valenta C, Auner BG. The use of polymers for dermal and transdermal delivery. Eur J Pharm Biopharm. Netherlands; 2004;58:279–289.
